# The combination of baseline magnetic resonance perfusion-weighted imaging-derived tissue volume with severely prolonged arterial-tissue delay and diffusion-weighted imaging lesion volume is predictive of MCA-M1 recanalization in patients treated with endovascular thrombectomy

**DOI:** 10.1007/s00234-013-1310-2

**Published:** 2013-12-15

**Authors:** F. Nicoli, F. Scalzo, J. L. Saver, F. Pautot, A. Mitulescu, Y. Chaibi, N. Girard, N. Salamon, D. S. Liebeskind, for the UCLA Stroke Investigators

**Affiliations:** 1 Medical and Research & Innovation Departments, Olea Medical, 93 avenue des sorbiers, ZI Athelia IV, 13800 La Ciotat, France; 2 UCLA Stroke Center, Los Angeles, CA USA; 3 Department of Neuroradiology, La Timone University Hospital, Marseille, France

**Keywords:** Stroke, MRI, Perfusion, Thrombectomy, Bayesian

## Abstract

**Introduction:**

Indices of collateral flow deficit derived from MR perfusion imaging that are predictive of MCA-M1 recanalization after intravenous thrombolysis have been recently reported. Our objective was to test the performance of such MRI-derived collateral flow indices for prediction of recanalization after endovascular thrombectomy.

**Methods:**

Fifty-seven patients with MCA-M1 occlusion evaluated with multimodal MRI prior to thrombectomy were included. Bayesian processing allowed quantification of collateral perfusion indices like the volume of tissue with severely prolonged arterial-tissue delay (>6 s) (VolATD6). Baseline DWI lesion volume was also measured. Correlations with angiographic collateral flow grading and post-thrombectomy recanalization were assessed.

**Results:**

VolATD6 < 27 ml or DWI lesion volume <15 ml provide the most accurate diagnosis of excellent collateral supply (*p* < 0.0001). The combination of VolATD6 > 27 ml and DWI lesion volume >15 ml significantly discriminates recanalizers versus nonrecanalizers (whole cohort, *p* = 0.032; MERCI cohort (*n* = 50), *p* = 0.024). When both criteria are positive, 76.2 % of the patients treated with the MERCI retriever do not fully recanalize (*p* = 0.024). In multivariate analysis, the aforementioned combined criterion and the angiographic collateral grade are the only independent predictors of recanalization with the MERCI retriever (*p* = 0.015 and 0.029, respectively).

**Conclusion:**

Bayesian arterial-tissue delay maps and DWI maps provide a non-invasive assessment of the degree of collateral flow and a combined index that is predictive of MCA-M1 recanalization after endovascular thrombectomy. Further studies are needed to evaluate the accuracy of this index in patients treated with novel stent retriever devices.

## Introduction

Several studies have demonstrated that better collateral flow is associated with a higher recanalization rate after endovascular therapy [1, 2]. In addition, recanalization status represents the strongest predictor of clinical outcome in patients after thrombectomy [3]. Given that the current reference method to evaluate the quality of collateral perfusion is the angiography performed just before thrombectomy, non-invasive methods are needed to predict recanalization and may also facilitate selection of acute ischemic stroke patients for endovascular therapy. Recent data from the literature suggest that multimodal MRI could be part of this strategy.

The presence of hyperperfusion on pulsed arterial-spin-labeling-type perfusion (ASL) is indicative of reperfusion/collateral flow in acute ischemic stroke. However, ASL provides lower signal intensity than conventional T2* imaging and ASL-derived CBF may not be sensitive to collateral flow above a given value [4]. Territorial ASL can also be used to noninvasively assess collateral circulation but is time-consuming and more complex to perform in the emergency management of an acute stroke [5]. In addition, to date, no ASL study aiming to assess collateral flow and predict recanalization after intravenous thrombolysis or endovascular thrombectomy has been reported in the literature.

More interestingly, Raychev et al. recently reported that the baseline volume of DWI abnormalities was an independent predictor of recanalization after thrombectomy in patients with MCA and ICA occlusion [6]. Furthermore, MR-PWI-derived indices of collateral circulation deficit have been recently described in patients with acute MCA-M1 occlusion. One, termed the nCCD index (normalized Collateral Circulation Deficit index), is based on Tmax maps at different time points and is calculated using a block-circulant SVD method [7]. The other, named VolATD6 and strongly correlated with nCCD, is based on arterial-tissue delay (ATD) maps accurately computed using a Bayesian algorithm and corresponds to the volume of tissue with severely prolonged ATD (>6 s) [8–10]. Indeed, in case of complete MCA-M1 occlusion, the ATD, defined as the difference in bolus arrival time between the arterial input function (AIF) and the tissue curve, is increased in relation with a slow retrograde blood delivery within the MCA territory during the arterial circulatory phase [8, 9, 11]. These parameters, i.e., nCCD and VolATD6, are both predictors of full MCA-M1 recanalization after IV thrombolysis and are highly reproducible [7, 9]. However, none of these indices have been tested yet to noninvasively predict MCA-M1 recanalization after endovascular thrombectomy.

The purpose of the current study is to test if MRI-derived collateral flow indices could predict MCA-M1 recanalization after thrombectomy.

As an initial step, a correlation study between the angiographic collateral grade (ACG) and baseline volume of tissue with prolonged ATD or DWI lesion volume was performed in patients with acute MCA-M1 occlusion evaluated with MR-PWI prior to angiography for thrombectomy.

Then, thresholds of tissue volume with prolonged ATD or DWI lesion volume that optimally diagnose very good to excellent collateral flow (angiographic collateral grade 3–4) were determined. Moreover, the ability of the most accurate parameters, alone or in combination, to differentiate recanalizers versus non recanalizers was analyzed in the whole cohort and in the subpopulation of patients treated with MERCI retriever (Concentric Medical) only.

Finally, a multivariate analysis was performed to determine if most discriminative parameters were independent predictors of MCA recanalization after thrombectomy with the MERCI retriever.

## Methods

### Study design

Fifty-seven patients with acute MCA-M1 occlusion were included in the study (Table 1). This cohort has already been reported in a recent correlation study between MR-PWI-derived collateral flow indices and the ACG [9]. These cases were selected from a cohort of patients with acute MCA-M1 occlusion evaluated with MR-PWI prior to angiography for thrombectomy and archived in a prospective single center registry. Demographic, clinical, laboratory, and imaging data were prospectively collected from 2003 to 2011 on consecutive patients who received endovascular therapy for acute cerebral ischemia. Institutional Review Board approval was obtained for these analyses on collateral perfusion. All selected patients have satisfactory-quality PWI records available for analysis. ACG was performed blindly to clinical data by an expert reader and evaluated with the American Society of Interventional and Therapeutic Neuroradiology/Society of Interventional Radiology Collateral Flow Grading System on baseline angiography [1]. Using this angiographic scale, patients are assigned to Grade 0 (no collaterals visible to the ischemic site), 1 (slow collaterals to the periphery of the ischemic site with persistence of some of the defect), 2 (rapid collaterals to the periphery of ischemic site with persistence of some of the defect and to only a portion of the ischemic territory), 3 (collaterals with slow but complete angiographic blood flow of the ischemic bed by the late venous phase), and 4 (complete and rapid collateral blood flow to the vascular bed in the entire ischemic territory by retrograde perfusion). Patient population was divided into recanalizers and nonrecanalizers depending on the degree of MCA-M1 recanalization after endovascular therapy (recanalizers = arterial occlusive lesion (AOL) 3 recanalization, i.e., full recanalization; nonrecanalizers = AOL 0–2 recanalization, i.e., partial or null recanalization [12, 13]). The degree of MCA recanalization was angiographically assessed at the end of the endovascular procedure.

**Table 1:** Patient population features and baseline characteristics (mean (SD))

	Intra-arterial therapy (*n* = 57)
Age	65.8 (18)
Sex ratio (M/F)	23/34
Diabetes (%)	24.6
Stroke etiology (CE/Atherom/Other) (%)	57.9/21/21
Onset-to-treatment time (min)	348.3 (178.1)
Baseline NIHSS	16.3 (5.7)
Onset-to-MRI time (min)	270.6 (166.7)
MRI-to-angiography time (min)	77.7 (41.6)
Baseline Vol DWI (ml)	34.3 (31)
Proximal MCA-M1 occlusion (%)	57.9
Distal MCA-M1 occlusion (%)	26.3
Tandem occlusion (%)	15.8
Combined IVT-IAT (%)	36.8
Thrombectomy (%)	100
Exclusive use of the MERCI retriever (%)	87.7
Full MCA-M1 recanalization during IAT (%)	47.4

*CE* cardioembolic etiology

### Image acquisition and processing

MRI of patients treated by endovascular route were acquired on a 1.5-T (Magnetom Vision+, Sonata or Symphony) or 3-T (Magnetom Trio) MRI (Siemens Medical Systems, Erlangen, Germany).

Diffusion-weighted images and MR perfusion images were processed blindly to clinical data and to ACG, using a development version of OleaSphere™ (Olea Medical, La Ciotat, France). Diffusion was measured at three values of b (*b* = 0, 500, 1,000 s/mm^2^). Average ADC maps were also generated. The volume of diffusion anomalies was quantified from analysis of isotropic b1000 DW images and ADC maps.

Perfusion-weighted imaging (PWI) was performed using an axial dynamic gradient-echo echo-planar perfusion-weighted sequence (TR mean value of 2.12 ± 0.29 s). After automatic selection of the AIF [7], a systematic check of the result was performed to be sure that the earliest AIF was automatically selected in each case. The pre-bolus baseline range was adjusted to reach the beginning of the AIF ascending slope. To increase the numerical accuracy of the Bayesian estimation of hemodynamic parameters (CBF, CBV, MTT, TTP, ATD), the step of the time grid used for the Bayesian computation of the residue function was increased, by introducing extra points between sampling time points, to reach a minimum temporal resolution of 0.5 s [10]. The mathematical algorithm of the Bayesian post-processing of MR-PWI data used in the current study has been previously detailed in Ref. [10].

Pixels corresponding to the CSF were automatically excluded using a brain tissue mask calculated from ADC maps and large vessels were removed using a maximum CBV threshold value of 7 or 8 ml/100 g, at the discretion of the operator’s visual analysis.

### Calculation of the Bayesian arterial-tissue delay maps

The Bayesian ATD is the time-to-maximum of the computed Bayesian residue function *R*(*t*) [10]. The Bayesian method is a robust probabilistic method for estimating the residue function of brain tissues, *R*(*t*), and hemodynamic parameters [14]. The Bayesian algorithm minimizes effects of oscillation, tracer delay and low SNR during *R*(*t*) estimation when compared with SVD methods [10, 14]. Indeed, severe overfitting and oscillations induce a systematic underestimation of the first values of the SVD-computed residue function which is responsible for the SVD overestimation of the delay [15]. Conversely, the Bayesian algorithm produces smoother estimate of the residue function and allows to accurately measure delays between the AIF and the tissue curve [10, 15]. In addition, in agreement with recommendations from the Acute Stroke Research Imaging Roadmap II [16], simulations were performed on MR digital phantom and confirmed that the ATD estimation by means of the aforementioned Bayesian method is highly accurate, even with a relatively high sampling time TR = 2 s as in the current study [10]. Besides, it is much more accurate by comparison with the oSVD method (SVD with oscillation index) [10]. These simulations also demonstrated only a slight overestimation of the AIF-tissue delay by the Bayesian method, in case of strong dispersion only [10].

The whole volume of tissue with increased ATD, a priori defined as the ipsilateral volume of tissue with ATD value > contralateral hemispheric ATD mean value + 1.5XATD standard deviation value, was measured (VolATD + 1.5SD). Residual artifacts persisting after this ATD thresholding procedure were manually excluded.

The volume of tissue with ATD > 6 s (VolATD6) was also determined to evaluate the severity of the collateral flow deficit [8, 9]. The inter-rater reproducibility of this volume measurement, assessed in a purposefully designed three-rater study on 25 cases with acute MCA-M1 occlusion, is very high (ICC(95 %CI) = 0.994(0.988–0.997)) [9]. Measurements of VolATD6 were automatically performed, without manual removal of residual artifacts, since an ATD threshold value as high as 6 s almost excludes the probability to include residual artifacts in the volume of interest [9].

Measurements of VolATD + 1.5SD and VolATD6 were performed on the whole brain.

### Measurement of baseline DWI lesion volume

The aim of this measurement was to determine the global impact of acute hypoperfusion on brain tissue as detectable by diffusion imaging. Thereby, the ischemic lesion was segmented from ADC and isotropic B1000 DWI maps and the highest lesional volume of the two was considered [7].

As an initial step, ADC threshold was set to 600 × 10^−6^ mm^2^/s [17] then, this threshold was manually adjusted based on visual information from B1000 images. Also ipsilateral and contralateral isolated artifacts were removed only upon visual control with regard to the B1000 image.

The inter-rater reproducibility of DWI lesion volume measurements was assessed in a purposefully designed 3-rater study on 48 patients with MCA-M1 occlusion already reported in Ref. [7]. Three raters (one neuro-imaging expert and two non-expert raters, fully blinded to clinical data) post-processed all images. Pearson correlation tests were used to establish inter-rater correlations. Intraclass correlation coefficients (ICC) were calculated to establish the level of inter-rater agreement. The standard deviation (SD) of differences between all raters’ measurements was also calculated to assess volume measurement variability, as follows. First, all raters (*r* = 1:3) perform one volume measurement (Vrp) for each patient (*p* = 1:48). Since no gold standard is provided, the mean of measures performed by the three raters was calculated for each patient (mVp), followed by the difference of each measure to the corresponding mean (dVrp = Vrp − mVp). SD on all these differences can be calculated to assess interoperator uncertainty [17]. 2SD is considered to be the confidence interval width at 95 %. In this study, only the SD value was used to simplify comparison with literature.

The inter-rater agreement for the lesion volume measurement is perfect, whatever the rater’s expertise and the correlations between raters’ measurements were also high (*r* ^2^ ≥ 0.74) (Table 2). In addition, the SD of the differences in volumes calculation between all three operators was 3.81 ml. These results are close to those of Ay et al. [18] and Luby et al. [19] who reported standard deviations of differences between two measurements of DWI lesion (between examiners) of 5.1 ml and 5.41 to 7.2 ml respectively.

**Table 2:** Intra-rater reproducibility results for DWI lesion volumes calculation

Statistical tests	Expert vs non-expert 1	Expert vs non-expert 2	Non-expert 1 vs non-expert 2	All raters
ICC (95 % CI)	0.90(0.83–0.94) ↑↑	0.78(0.64–0.87) ↑	0.92(0.87–0.95) ↑↑	0.86(0.79–0.91) ↑↑
r2	0.86	0.74	0.86	NA

*↑↑* ICC value indicates perfect inter-rater agreement, *↑* ICC value indicates strong inter-rater agreement, *NA* not applicable

### Statistical analysis

The statistical analysis was performed blindly to clinical data. All statistical tests were performed by means of JMP-9* software (SAS Institute Inc., Cary, NC, USA).

The strength of the relationship between continuous quantitative variables like MR-PWI-derived parameters and a discrete ordinal variable like ACG was measured by means of the Spearman’s rho correlation coefficient.

The comparison of group size was performed using either a two-tail Fisher’s exact test for two statistical populations or a Pearson’s chi square combined for three statistical populations. The strength of the association between variables significantly correlated according to the Pearson’s chi square test was measured using the Cramer’s V contingency coefficient.

The Gaussian distribution of tested parameters was assessed by a Shapiro–Wilk test.

A ROC analysis was performed to determine optimal thresholds of Bayesian hemodynamic parameters providing the best discrimination between angio grades 0–2 versus 3–4. A ROC curve was generated for each Bayesian hemodynamic parameter. For each curve, the area under the curve (AUC) and optimal operating point were determined. The optimal operating point, that determines the optimal threshold for a given hemodynamic parameter, is the probability threshold that gives the smallest false-positive rate (1 − specificity) for the largest true-positive rate (sensitivity). The Youden’s index, defined as sensitivity + specificity − 1, was also used to compare the discriminative performance of each optimal threshold.

A multivariate analysis was performed using multiple logistic regressions to determine the independent predictors of MCA recanalization after thrombectomy. The results were validated by means of a goodness-of-fit test. The statistical significance threshold was set to *p* < 0.05. A statistical trend was defined as *p* < 0.15.

## Results

**Fig. 1:**
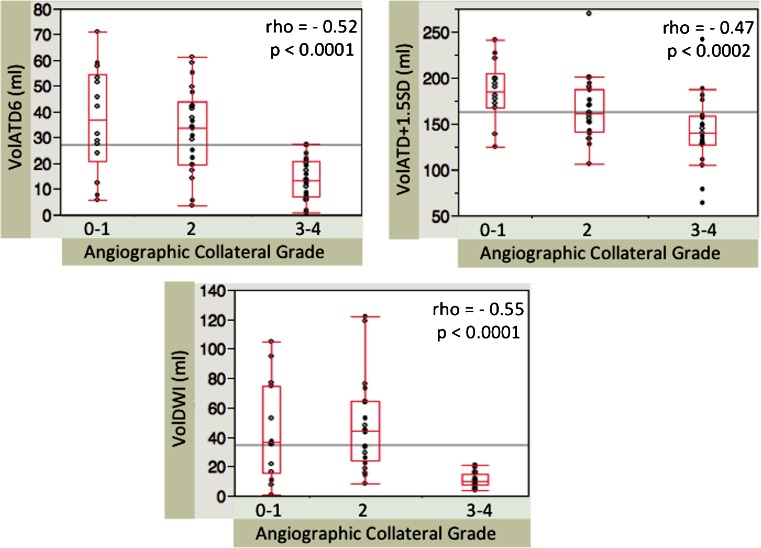
Correlations between Bayesian parameters, diffusion lesion volume and the angiographic collateral grading (0–1 vs 2 vs 3–4) in the whole cohort. VolATD6 and VolDWI have the highest correlation with the angiographic collateral grading. More extensive collaterals significantly correlate with smaller baseline volume of tissue with severely increased ATD and smaller baseline DWI lesion volume (Spearman’s rank correlation)

Details on patient population features and baseline characteristics are presented in Table 1. Most of the patients were treated with the MERCI retriever. Five patients were treated with the PENUMBRA system (Penumbra Inc.) and only one with the SOLITAIRE FR device (Covidien). Combined IV and IA therapy was performed in about 37 % of the cases. Full MCA-M1 recanalization (AOL 3) was achieved in 47.4 % of the cases. In the current series, 40.3 % of the patients were included in a trial and had a documented mRS at 3 months (56.5 % recanalizers, 43.5 % non recanalizers). At 3 months, 53.8 % of this subset of recanalizers and 20 % of this subset of non recanalizers were functionally independent (mRS 0–2). In the whole cohort, four patients were assigned to ACG 0, 10 to grade 1, 22 to grade 2, 14 to grade 3 and 7 to grade 4. Considering the small sample size of grades 0 and 4 and to improve the accuracy of the statistical comparison between groups, patients included in angiographic collateral grades 0 and 1 (very bad collateral flow) were assigned to one group named ACG 0–1 and patients included in angiographic collateral grades 3 and 4 (very good collateral flow) were assigned in another group named ACG 3–4. Therefore, the statistical analysis was based on an ACG with only three levels: 0–1 versus 2 versus 3–4 (Fig. 1).

A ROC analysis determined that optimal thresholds of prolonged ATD tissue volume providing the best discrimination between angio grades 0–2 versus 3–4 were: VolATD6 threshold = 27 ml (AUC = 0.84; sensitivity = 100 %; specificity = 67 %; Youden’s index = 0.67) and VolATD + 1.5SD threshold = 159 ml (AUC = 0.75; sensitivity = 81 %; specificity = 67 %, Youden’s index = 0.48). A pre-treatment DWI lesion volume value of 15 ml provides a clear-cut discrimination between angiographic grades 0–2 versus 3–4 on box-plot analysis (sensitivity = 80 %; specificity = 85.7 %; Youden’s index = 0.66) (Fig. 1).

VolATD6 and VolDWI optimal thresholds tend to more accurately discriminate patients with very good collateral flow and to better discriminate recanalizers versus non recanalizers when compared with optimal threshold of VolATD + 1.5SD (Fig. 1, Tables 3 and 4).

**Table 3:** Discrimination between grades 0–2 versus 3–4 in the whole cohort

	Grades 0–2 versus 3–4
	*p* value
VolATD6 > 27 ml	<0.0001
VolATD + 1.5SD > 159 ml	0.0008
VolDWI > 15 ml	<0.0001

**Table 4:** Discrimination between recanalizers versus non recanalizers in the whole cohort and in the subpopulation of patients treated with the MERCI retriever only

	Whole cohort (*n* = 57)	MERCI cohort (*n* = 50)
	*p* value^a^	*p* value^a^
VolATD6 > 27 ml	0.111	0.093
VolATD + 1.5SD > 159 ml	0.113	0.153
VolDWI > 15 ml	0.056	0.121
VolATD6 > 27 ml and DWI > 15 ml	0.032	0.024
Angio grade 0–1 vs 2–4	0.0054	0.011
Angio grade 0–2 vs 3–4	0.0069	0.015

^a^Two-tailed Fisher’s exact test

The angographic collateral grading and the combination of VolATD6 and DWI lesion volume measurements significantly discriminate recanalizers from nonrecanalizers, whether patients are treated with the MERCI retriever only or not (Table 4). When the VolATD6 > 27 ml and DWI > 15 ml combined criterion is positive in patients treated with the MERCI retriever only, 76.2 % of the patients do not fully recanalize (16/21) and 100 % of the patients are assigned to angiographic grades 0–2 (absent to moderate collateral circulation) (21/21) while 71 % are assigned to angiographic grades 0–1 (absent to very poor collateral circulation) (10/14).

When this criterion is negative, 57.1 % of the patients fully recanalize (16/28) and 85.7 % of the patients are assigned to groups 2–4 (moderate to excellent collateral circulation) (24/28) while 57.1 % are assigned to angiographic grades 3–4 (very good to excellent collateral circulation) (10/14).

By comparison, in patients treated with the MERCI retriever only, the angiographic grades 0–1 significantly discriminate recanalizers versus nonrecanalizers. When patients are assigned to these grades, 85.7 % of the patients do not fully recanalize (12 over 14). Conversely, if patients are assigned to grades 2–4, 55.5 % of the patients fully recanalize (20 over 36).

### Multivariate analysis

Since the recanalization rate depends on the retrieval device used for thrombectomy [20, 21], this analysis was performed on the MERCI cohort only (*n* = 50).

The multiple logistic regression analysis including the angiographic collateral grade 0–2, the use of a combination therapy, the presence of a tandem occlusion and the time-to-IA therapy higher than 240 min shows that the ACG is the only independent predictor of MCA recanalization after thrombectomy with the MERCI retriever (goodness-of-fit test, *p* = 0.745; odds ratio 0.21 95 % CI 0.050–0.753; Wald test, *p* = 0.029).

The multiple logistic regression including the VolATD6 > 27 ml and DWI > 15 ml combined criterion, instead of the ACG, and the use of a combination therapy, the presence of a tandem occlusion and the time-to-IA-therapy higher than 240mn shows that the combined MR criterion is also the only independent predictor of MCA recanalization after thrombectomy with the MERCI retriever (goodness-of-fit test, *p* = 0.963; odds ratio 0.175 95 % CI 0.038–0.670; Wald test, *p* = 0.015).

The inclusion of VolATD6 > 27 ml and DWI > 15 ml as two separate dichotomized variables and of their interaction, instead of the MRI combined criterion within the multivariable logistic model, worsens this model.

In these multiple regression analyses, the choice to include the dichotomized parameter time-to-IA-therapy > 240 min instead of the continuous corresponding variable is firstly due to its non Gaussian and non normalizable distribution and secondly to the reported better outcome in patients who recanalize after thrombectomy within 300 min after stroke onset [22]. The difference of 60 min between these two delays corresponds to an estimation of the mean time needed to achieve per-procedure MCA recanalization.

In addition, questioning a bias related to a possible link between the year of the endovascular procedure and the MCA recanalization rate, during the 9-year inclusion period, the year of the thrombectomy was entered into these multivariate analyses but does not modify their conclusions.

## Discussion

Previous studies already demonstrated that a non-invasive collateral flow assessment is able to predict MCA-M1 recanalization after IV thrombolysis [7–9].

The current study demonstrates that a similar prediction is possible in patients undergoing endovascular thrombectomy. Indeed, in this series, the criterion combining baseline DWI lesion volume and volume of tissue with arterial-tissue delay over 6 s is significantly correlated with the angiographic collateral grading, and significantly discriminates recanalizers from non recanalizers after thrombectomy with the MERCI retriever.

This is the first step toward a non-invasive prediction of recanalization after thrombectomy. It provides the potential opportunity to improve the penumbral imaging-based selection of patients for thrombectomy.

Indeed, the negative results of all the recent therapeutic trials for thrombectomy not only suggest that the development of more effective endovascular devices is imperative but also that better selection of the patients could be a new strategy for future trials [23–26]. The IMS III and SYNTHESIS Expansion studies, that did not use mismatch-based imaging selection of patients, show that intravenous thrombolysis is still the first-line treatment within 4.5 h after ischemic stroke onset, even if imaging shows an occluded major intracranial artery [23, 24]. Beyond 4.5 h, the MR RESCUE trial, whose sample size is relatively small, does not provide data supporting the use of endovascular treatment in patients with an ischemic penumbra as defined in this study [25]. However, all these trials used older retrieval devices with a significantly lower recanalization rate compared with recent stent retrievers [20, 21]. Thus, the ischemic penumbra hypothesis tested in MR RESCUE should be retested in a larger randomized trial with more efficient recent retrievers [26]. Moreover, a recent study also finds another explanation for negative results in the MR RESCUE study. Indeed, authors of this study demonstrated that, in patients treated with IV thrombolysis and/or endovascular thrombectomy, prediction of tissue fate using CT-based penumbral imaging cannot predict clinical outcome without combining prediction of recanalization [27]. Similarly, patients with a MRI-derived target mismatch profile, hallmarking penumbra, have a more favorable clinical outcome if reperfusion is achieved after thrombectomy [28]. However, if recanalization is not achieved, the penumbral information becomes somewhat futile.

Thus, the prediction of recanalization and ultimately reperfusion is becoming a new challenge in the field of non-invasive multimodal imaging aimed to improve the selection of patients for recanalization therapies [27].

### Choice of the angiographic revascularization grading

In the SWIFT randomized controlled trial, a successful recanalization was defined as TICI 2 or greater reperfusion (Thrombolysis In Cerebral Infarction) [20]. However, in the current study, the AOL scoring was preferred to evaluate the arterial patency at the end of the procedure. AOL and TICI scores are strongly correlated [13]. Moreover, measures performed after the end of the thrombectomy procedure in the SWIFT trial, including any rescue therapy, demonstrated that AOL 2–3, TIMI 2–3 and TICI 2b–3 scores did not differ significantly in predicting good neurological outcome (mRS 0–2 or equal to baseline mRS if baseline mRS was greater than 2, or improvement of at least 10 points in NIHSS score) (57, 62.9, 61.8 %, respectively; AOL versus TIMI *p* = 0.31, AOL versus TICI *p* = 0.095) [13].

In addition, in the SWIFT study, ratios of successful recanalization rate over good neurological outcome rate are very close in patients treated with the SOLITAIRE device versus those treated with the MERCI device (1.19 (69/58) and 0.91 (30/33), respectively) [20]. Thus, the prediction of a good clinical outcome using the recanalization criterion seems independent of the thrombectomy device used which highlights the interest of this prediction in the selection of patients for thrombectomy. The aim of the current study is to perform a non invasive prediction of recanalization through the assessment of the collateral flow in patients mostly treated with the MERCI retrieval device.

### Correlations between MRI-derived parameters and the angiographic collateral flow grading

All tested parameters are significantly correlated with the ACG. However, VolATD6 and baseline DWI lesion volume perform best (Fig. 1). Moreover, their corresponding optimal thresholds tend to more accurately detect patients with excellent collateral flow compared with optimal threshold of VolATD + 1.5SD (Table 3). The Youden’s index calculation confirms higher discriminative performances of VolATD6 and VolDWI thresholds by comparison with VolATD + 1.5 threshold (Youden’s index value = 0.67, 0.66, 0.48 respectively). Trends to discriminate recanalizers versus non recanalizers are also better for VolATD6 and baseline DWI lesion volume (Table 4). Therefore, only the combination of these two parameters was tested in an attempt to improve the MRI prediction of MCA recanalization after thrombectomy.

### Predictive value of baseline DWI lesion volume on MCA recanalization

Raychev et al. recently reported that the baseline volume of DWI abnormalities was an independent predictor of recanalization after thrombectomy in a cohort of 105 patients with ICA and MCA occlusion treated with multimodal mechanical device strategies (MERCI ± Penumbra ± angioplasty and stenting) (logistic regression analysis, OR = 0.238, *p* = 0.046) [6].

In the current series including a smaller cohort of 57 patients with MCA ± ICA occlusion mostly treated with the MERCI retriever ± IVT, the baseline DWI lesion volume threshold >15 ml, accurately discriminating angio grades 0–2 versus 3–4, only shows a trend to discriminate recanalizers versus non recanalizers (Fisher’s exact test, *p* = 0.056 in the whole cohort) (Table 4).

Similarly, Olivot et al. [29] reported that patients with full recanalization after endovascular therapy for MCA and/or ICA occlusion (SNARE (eV3) or SOLITAIRE (Covidien) devices, ±combined IV or IA rtPA) tend to have a smaller baseline median DWI lesion volume versus patients with partial or no recanalization (10, 21, and 19 ml respectively; *p* = 0.07). In this series, the largest DWI lesion volume associated with a favorable outcome in non recanalizers was relatively small and equal to 20 ml [29]. Although Olivot et al. did not report the collateral flow status in these patients [29], their observations are in agreement with the significant relationship between the baseline DWI lesion volume and the ACG demonstrated in the current study. Indeed, one could expect that patients with MCA occlusion associated with an excellent collateral flow, as suggested by a small baseline DWI lesion volume, might have a favorable outcome in spite of a persisting MCA occlusion.

Interestingly, in patients with similar arterial occlusions treated with IVT, Nicoli et al. [7] and Nighogossian et al. [30] reported a significantly smaller initial DWI lesion volume in recanalizers (mean value = 13 ml and median value = 13 ml, respectively) versus non recanalizers (mean value = 23.4 ml and median value = 48 ml, respectively).

Thus, whether MCA recanalization is obtained after IVT [7, 30] or thrombectomy [29] recanalizers have a relatively small baseline DWI lesion volume (# <20 ml). In addition, this range of baseline DWI lesion volume is significantly correlated with very good collateral perfusion.

### Predictive value of Bayesian MR-PWI-derived collateral flow index on MCA recanalization

The positive impact of the degree of collateral flow on MCA recanalization after thrombectomy has already been demonstrated [1, 2]. However, in the current series, despite a significant correlation with ACG and its very good diagnostic accuracy for discriminating patients with or without very good collateral flow (AUC = 0.84), the baseline volume of tissue with ATD > 6 s. only shows a nonsignificant trend to predict MCA M1 recanalization after thrombectomy (Fisher’s exact test, *p* = 0.111 in the whole cohort, *p* = 0.093 in the MERCI cohort) (Table 4); whereas the collateral grading alone, performed later during angiography, is able to accurately discriminate recanalizers from nonrecanalizers. Further investigations are required to determine if the discrepancy between the prediction of MCA recanalization by VolATD6 versus ACG is related to an insufficient accuracy of the PWI-based collateral flow assessment, and/or to fluctuations in hemodynamic conditions and collateral supply during the MRI-to-angiography time that would decrease the predictive value of the VolATD6 measurement on later ACG and recanalization.

### Added value of DWI and PWI to predict recanalization after thrombectomy

In the current series, the combination of VolATD6 and DWI lesion volume measurements significantly discriminates recanalizers from nonrecanalizers, whether patients are treated with the MERCI retriever only or not (Table 4). These findings suggest that these two parameters, significantly correlated with the ACG, provide complementary and non redundant information about the degree of collateral flow and its ability to increase the full MCA recanalization rate. The highest sensitivity of VolATD6 optimal threshold (100 %) combined with the highest specificity of VolDWI optimal threshold (85.7 %) for diagnosing very good collateral flow may contribute to the better prediction of full MCA-M1 recanalization. Indeed, the post hoc analysis of the correlation between these two MR-derived collateral flow indices and the ACG (0–1 vs 2 vs 3–4) demonstrates that this correlation with the ACG is higher for the combined criterion (VolATD6 > 27 ml: Pearson’s chi square = 12.75, Cramer’s *V* = 0.47, *p* = 0.0017; DWI > 15 ml: Pearson’s chi square = 13.92, Cramer’s *V* = 0.49, *p* = 0.0009; combined criterion: Pearson’s chi square = 18.42, Cramer’s *V* = 0.57, *p* < 0.0001). In addition, at the time of the MRI examination, the VolATD6 measurement provides an instantaneous estimation of the degree of collateral flow while the DWI lesion volume corresponds to a tissue marker for the efficiency of collateral perfusion to preserve tissue from ischemia during the time to MRI. Thus, the combined criterion increases the MRI information content on the degree of collateral flow and facilitates discrimination of recanalizers and nonrecanalizers.

Moreover, like the angiographic collateral grading, this combined criterion is an independent predictor of MCA recanalization after thrombectomy with the MERCI retriever.

### Impact of the combined therapy on post-thrombectomy recanalization

In patients with MCA-M1 occlusion treated with IVT within the 3-h time window, the vigor of the collateral flow is predictive of a full MCA recanalization [7]. Beyond 3 h, collateral circulation does not significantly potentiate the action of IV tPA on the clot, probably because the longer a clot persists over time, the more resistant to fibrinolysis it becomes [7]. However, even when failing to recanalize the occluded artery, IVT performed before thrombectomy could be able to (1) limit the thrombus extension by delivering rtPA to proximal and distal parts of the clot via efficient collaterals [7] and (2) make the mechanical thrombectomy easier thanks to a relatively lower clot size compared with thrombectomy alone.

Thus, the relationship between collateral flow and the success of the thrombectomy procedure could only be driven by IV rtPA. Interestingly, this bridging therapy was applied in 33 to 58 % of the cases treated with a stent retriever in SWIFT and TREVO II trials [21].

However, in the current series, only 16 patients were treated with IVT and the MERCI retriever making difficult to definitely conclude about the rational for the influence of the vigor of the collateral flow on MCA recanalization in patients treated with bridging therapy versus thrombectomy alone. The frequency of this combined therapy was not statistically different in recanalizers versus nonrecanalizers (31.8 % (7/22) and 32.1 % (9/28) respectively). In addition, the multivariate analysis including the variable “combined therapy” demonstrates that only the ACG and the combined MR criteria are independent predictors of full MCA recanalization after thrombectomy with the MERCI retriever.

### How may the vigor of the collateral flow influence MCA recanalization in patients treated with thrombectomy alone?

The answer to this question raised by the results of the aforementioned multivariate analysis is more speculative and needs more investigations. Virchow’s triad is traditionally invoked to explain pathophysiologic mechanisms leading to thrombosis, alleging concerted roles for abnormalities in blood composition (e.g., platelet activation), endothelium injury, and reduced blood flow in the development of arterial and venous thrombosis [31]. Given this pathophysiology, one could suggest that in patients treated with thrombectomy alone, a better collateral flow decreases the relative importance of the reduced blood flow component of the Virchow’s triad within the MCA vascular bed, which could limit the thrombus extension and make the thrombectomy procedure easier.

Similarly, Jovin et al. had previously suggested that patients with M1 or ICA terminus occlusion and severely hypoperfused hemisphere have a higher proportion of ischemic core in which, because of stagnant flow, the vessels supplying the area have a higher clot burden in the vascular bed of the affected territory which decreases the probability of recanalization [32]. Interestingly, these authors demonstrated that the higher the regional CBF in the ipsilateral hemisphere, the higher the likelihood of successful intra-arterial thrombolysis [32]. Given the significant relationship between the ipsilateral rCBF and the collateral flow deficit index VolATD6 (linear regression, negative correlation, *r* ^2^ = 0.63; *p* < 0.0001) demonstrated in patients with MCA-M1 occlusion [8], the results from Jovin et al. suggest that a good collateral flow might also facilitate recanalization beyond 3 h during intra-arterial thrombolysis, thanks to a potential lower clot burden due to a reduced intravascular stagnant flow [7].

To sum up, the instantaneous estimation of the vigor of the collateral flow is predictive of MCA recanalization when performed just before the recanalization therapy, either by means of PWI before IV thrombolysis [7–9] or by means of angiography before thrombectomy [1, 2, current study].

By contrast, when the MRI-based collateral perfusion assessment is performed before the endovascular procedure, the time between this measurement and the reperfusion therapy is longer and the instantaneous estimation of the vigor of the collateral flow using a PWI-derived index is no longer effective alone to predict recanalization after a thrombectomy performed later.

However, if this index is combined with the baseline DWI lesion volume, predictions of the delayed angiographic collateral grading and of post-thrombectomy recanalization are both significantly improved.

The baseline DWI lesion volume has a strong prognostic value for dependency, death and intracerebral hemorrhage in patients with acute ischemic stroke including those who underwent endovascular treatment [29, 33, 34]. It should also be considered as an index of average reactivity of the collateral supply in acute MCA M1 occlusion with potential hemodynamic fluctuations during the onset-to-MRI time. Thus, it might be more informative than a simple instantaneous measurement of the collateral flow efficiency and an important complementary functional parameter to take into account in a MRI-based collateral supply assessment.

### Limitations of the study

The first limitation is the retrospective design of the study. However, patients analyzed in this series have been included in a prospective clinical registry from one stroke center with consistent and standardized medical care.

The 9-year inclusion period could be responsible for learning-curve related biases, such as a higher recanalization rate due to increased thrombectomy experience in the stroke center. However, the multivariate analysis demonstrated that this parameter was not an independent predictor of recanalization.

Another limitation is the time elapsed between the MR examination and the angiographic exploration. Indeed, fluctuations in the degree of collateral flow may occur and impact the correlations between early MR-PWI collateral flow assessment and the later angiographic collateral grading [35, 36]. Although the relatively short mean MRI-to-angiography time (Table 1) minimizes such bias, further prospective study is warranted, also with a particular caution about the systematic registration of hemodynamic fluctuations occurring during the MRI-to-angiography time.

In addition, the baseline DWI lesion volume threshold that accurately identifies patients with excellent collateral flow in the current study must not be extrapolated to patients with MCA-M1 occlusion treated within a shorter onset-to-treatment time. Indeed, within 3 h after stroke onset, the baseline DWI lesion volume might be small in spite of a weak collateral flow because the MRI was performed early during the infarct growth. Conversely, the same small baseline DWI lesion volume diagnosed beyond 3 h, a usual onset-to-endovascular thrombectomy time, argues for a slow infarct growth, more probably in relation with a good collateral flow.

Finally, the almost exclusive use of the MERCI retriever in the current study impedes us to extrapolate the predictive value of the combined criterion to predict MCA recanalization after endovascular thrombectomy using other devices. Indeed, it has been recently demonstrated that recanalization rate depends on the retriever used for thrombectomy [20, 21]. Therefore, these results need to be confirmed in a larger cohort of patients having undergone thrombectomy with different retrievers.

## Conclusions

The current study demonstrates that MR-PWI and DWI can noninvasively assess the degree of collateral perfusion and predict the rate of full recanalization of MCA-M1 occlusions in patients treated with the MERCI retriever. Baseline DWI lesion volume and VolATD6 used in combination to achieve such prediction are highly reproducible. This combined criterion, closely correlated with the ACG, may improve and promote the non-invasive selection of patients for endovascular therapy, especially when coupled with a penumbral imaging able to select responders to recanalization. However, further studies are necessary to fully confirm our findings and evaluate the accuracy of this method to predict MCA recanalization with other models of retrieval devices.

## Abbreviations

ASL: Arterial-spin labeling
nCCD: Normalized collateral circulation deficit index
SVD: Singular value decomposition
oSVD: Singular value decomposition with oscillation index
VolATD6: Volume of tissue with arterial-tissue delay higher than 6 s
ATD: Arterial-tissue delay
AIF: Arterial input function
ACG: Angiographic collateral flow grading
AOL: Arterial occlusive lesion
TICI: Thrombolysis in cerebral infarction
